# *Diplodiscus latifii* (Malvaceae-Brownlowioideae), a new species from Sabah, Malaysia

**DOI:** 10.3897/phytokeys.161.55781

**Published:** 2020-10-02

**Authors:** Richard Cheng Kong Chung, Engkik Soepadmo

**Affiliations:** 1 Forest Research Institute Malaysia, 52109 Kepong, Selangor, Malaysia Forest Research Institute Malaysia Kepong Malaysia

**Keywords:** Borneo, conservation, flora, Malvaceae, morphology, taxonomy

## Abstract

A new species of *Diplodiscus* is described and illustrated from one collection made in lowland mixed dipterocarp forest in Sabah, Malaysia. Conspicuous by its twig colour, leaf shape and indumentum, it is probably allied to *D. longifolius*, but differs in sufficient characters (shape, indumentum, apex and venation of blade, flower bud shape and size, petal diameter, ovary shape and fruit shape and size) to be a species in its own right. A key to the species of *Diplodiscus* in Malaysia also is provided.

## Introduction

*Diplodiscus* Turcz. (Malvaceae-Brownlowioideae) is a genus of trees or shrubs, comprising nine species in Peninsular Malaysia, Borneo and the Philippines with its centre of distribution in Borneo (Kostermans 1960; Bayer and Kubitzki 2003; Mabberley 2017). In Peninsular Malaysia, the genus is currently represented by two species (i.e. *D. scortechinii* (King) Ashton ex Kochummen and *D. hookerianus* (King) Kosterm.), while in Borneo, it is represented by seven species (six endemic) and in the Philippines, it is represented only by the endemic type species (*D. paniculatus* Turcz.) (Kostermans 1960, 1965, 1992; Kochummen 1973; Ashton 1988; Turner 1997; LaFrankie 2010). One species, *D. scortechinii* is known to occur both in Peninsular Malaysia and Borneo (Sarawak and Brunei). In Borneo, *D. longifolius* (Merr.) Burret is found in Sabah and Sarawak, while *D. parviflorus* Kosterm. and *D. microlepis* Kosterm. are reported from Sabah and *D. aureus* Kosterm., *D. decumbens* Kosterm. and *D. longipetiolatus* Kosterm. are known to occur in Kalimantan (Kostermans 1960, 1965, 1992).

Two species, formerly in *Diplodiscus*, namely *D. verrucosus* (Thwaites) Kosterm. of Sri Lanka (Kostermans 1961) and *D. trichospermus* (Merr.) Y.Tang, M.G.Gilbert and Dorr of Guangxi and Hainan, China (Tang et al. 2007) are now in *Pityranthe* Thwaites (Malvaceae-Brownlowioideae) (Bayer and Kubitzki 2003; LaFrankie 2010; Christenhusz et al. 2017).

Our ongoing revision of the genus for the Flora of Peninsular Malaysia and the Tree Flora of Sabah and Sarawak recognises two species of *Diplodiscus* from Peninsular Malaysia and eight from Borneo, respectively. Of these, *D. latifii*, endemic to Sabah, is new to science and is here described for the first time as a precursory paper to the publication of a revision of the genus.

## Materials and methods

The morphological descriptions and comparisons are based on observations of specimens in a number of herbaria (BM, BO, K, KEP, L, SAN, SAR and SING; herbarium acronyms follow Thiers 2020) using Olympus SZ61 and LEICA M125 stereomicroscopes. The dimensions given in the descriptions are from dried material except that gynoecium and androecium characters are from rehydrated flowers. Terminology of trichome types mainly follows Webster et al. (1996).

## Taxonomic treatment

### Diplodiscus latifii

Taxon classificationPlantaeMalvalesMalvaceae

R.C.K.Chung sp. nov.

6BD2214B-9777-5B23-9F8D-DDCC16244251

urn:lsid:ipni.org:names:

Figures 1 , 2A–C

#### Diagnosis.

*Diplodiscus latifii* is closely related to *D. longifolius* in leaf margin, midrib, inflorescence position, flower number, calyx shape, androgynophore length and shape and fruit indumentum. However, the former differs from the latter by its dark brown to black twigs (vs. grey to light brown), elliptic to broadly elliptic leaves (vs. narrowly elliptic or obovate), densely covered in brown dentate-lepidote scales with scattered dark brown subentire-lepidote scales below (vs. densely covered in light brown stellate-lepidote scales), coriaceous leaf texture (vs. chartaceous), acute apex (vs. acuminate), densely dentate-lepidote scaled petioles (vs. densely light brown stellate-lepidote scaled or glabrescent), ellipsoid flower buds, 3.5–4 mm in diameter (vs. spheroid, 2–3 mm in diameter), ca. 2.5 mm width of petals (vs. ca. 4.5 mm width), ellipsoid ovary (vs. oblate) and ellipsoid fruits to 7 mm in diameter (vs. broadly ellipsoid and more than 10 mm in diameter). The main morphological differences between these two species are shown in Table 1.

**Figure 1.**
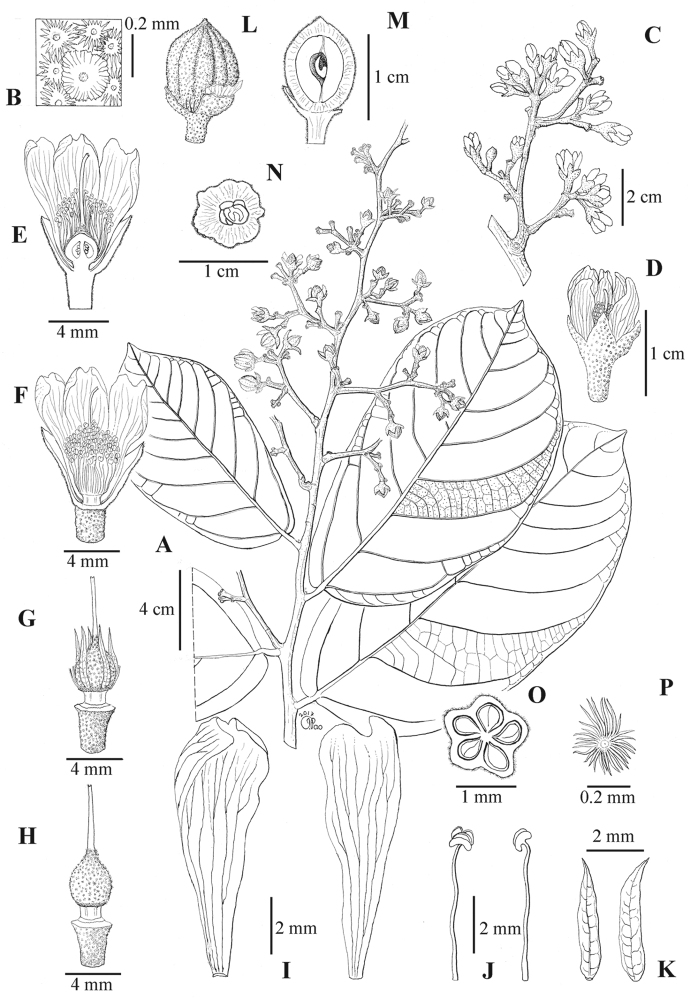
*Diplodiscus latifii***A** fruiting leafy twigs **B** leaf lower surface densely covered in dentate-lepidote scales with scattered subentire-lepidote scales **C** inflorescence **D** mature flower bud **E** longitudinal-section of flower **F** opened flower with parts of the calyx and two petals removed **G** flower with the calyx, petals and stamens removed **H** flower showing androgynophore and pistil **I** petals, abaxial view (right) and adaxial view (left) **J** stamens, lateral view (right) and slightly to top view (left) **K** staminodes, abaxial view (right) and adaxial view (left) **L** fruit with ridges and stellate trichomes **M** longitudinal-section of fruit **N** cross-section of fruit with one developed and four aborted seeds **O** cross-section at the centre of ovary **P** stellate trichome on the ovary surface. All drawn from the type. Drawn by Joseph Pao.

#### Type.

Borneo, Sabah, Lahad Datu, Silam, Mile 14, 11 January 1966, *Ahmad Talip SAN 52945* (holotype: SAN!; isotypes: K (sheet 1)!, K (sheet 2)!).

#### Description.

Small tree ca. 10 m tall, to 9 cm in diameter. ***Bark*** smooth, whitish; inner bark orange. ***Twigs*** rounded, 6–10 mm in diameter, glabrous and smooth, dark brown to black when dried. ***Stipules*** caducous. ***Leaves*** alternate, yellow brown with scattered tiny black dots below, coriaceous, densely covered in brown dentate-lepidote scales with scattered dark brown subentire-lepidote scales below, glabrous above; blades elliptic to broadly elliptic, (12.5–)15.5–20(–22) × (5.5–)8.5–10.5(–13) cm, base oblique, subcordate, margin entire, apex acute; midrib rounded, prominent below, raised above, glabrescent to glabrous below, glabrous above; lateral veins 8–10 pairs, prominent on both sides, looping near margin forming conspicuous intramarginal veins, basal pair of veins prominent on both sides, ascending to ¼ of the blade length; tertiary veins reticulate, prominent on both sides; petioles stout, transversally cracked, 7–14 mm long, 2–4 mm thick, densely covered in brown dentate-lepidote scales. ***Inflorescences*** terminal and axillary, panicles of 3–4(–5)-flowered cyme-like units, 15–23 cm long, 0.3–0.5 cm wide, lax, peduncles and rachis pale yellowish-brown, rounded, densely covered in stellate- and dentate-lepidote scales; bracts and bracteoles caducous, rarely persistent. ***Flowers*** bisexual, actinomorphic, pedicellate; buds ellipsoid or rarely spheroid, 4–5 × 3.5–4 mm, densely covered in stellate- and dentate-lepidote scales; pedicels 2–4(–5) × 1–2 mm, densely covered in stellate- and dentate-lepidote scales. Calyx bell-shaped, pale yellowish-brown; ca. 6 mm long; lobes 5, triangular, valvate, erect, 2–3 mm long, apex acute, densely covered in stellate- and dentate-lepidote scales abaxially, glabrous adaxially. Petals 5, white, spathulate, ca. 9 × 2.5 mm, base gradually tapering, apex emarginate, glabrous on both sides. Androgynophore short, cylindrical, ca. 1 mm long, glabrous. Staminodes 5, lanceolate, ca. 4 × 0.8 mm, shorter than filaments of fertile stamens, glabrous. Fertile stamens numerous; filaments arranged in 5 obscure phalanges, slightly connate at base, ca. 5 mm long, glabrous; anthers 0.6–0.8 mm in diameter. Ovary ellipsoid, ribbed, ca. 4 × 2.5 mm, densely stellate hairy, carpels 5, united, each carpel with 2 ovules; style 1, ca. 4.5 mm long, glabrous; stigma punctiform. ***Infructescences*** to 15 cm long, densely covered in stellate- and dentate-lepidote scales. ***Fruits*** ellipsoid, ribbed, 6–10 × 4–7 mm, densely stellate hairy; stalk ca. 2 mm long, ca. 1.5 mm thick, sparsely covered in stellate- and dentate-lepidote scales; pericarp ca. 1.5 mm thick. ***Seed*** 1(–2).

**Figure 2.**
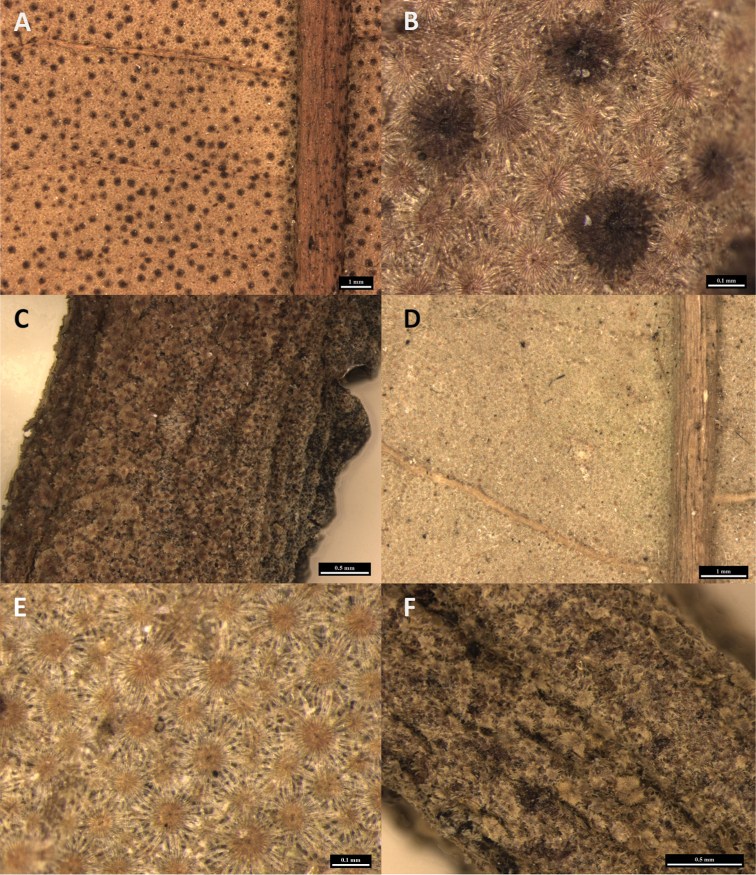
*Diplodiscus latifii* (**A–C**) and *D. longifolius* (**D–F**) **A** lower surface of leaves with scattered prominent black dots and densely small pale brown scales observed under low magnification **B** lower surface of leaves densely covered by brown dentate-lepidote scales with scattered dark brown subentire-lepidote scales **C** petiole densely covered by brown dentate-lepidote scales **D** lower surface of leaves densely covered by light brown stellate-lepidote scales observed under low magnification **E** leaves densely covered by light brown stellate-lepidote scales **F** petiole densely covered by light brown stellate-lepidote scales.

#### Distribution.

This species is endemic to Sabah, known from Silam, Lahad Datu.

#### Ecology.

Lowland mixed dipterocarp forest, yellowish soils, near stony stream at about 180 m elevation.

#### Vernacular names.

Pinggau-pinggau, takalis.

#### Etymology.

This species is named after the current Director-General of the Forest Research Institute Malaysia, Datuk Dr Abd. Latif bin Mohmod, for his strong support in conservation of karst limestone hills and plant diversity in Malaysia. He is the first and only forestry scientist ever honoured with a Malaysian National Young Scientist Award in 1993, two national science awards and six Malaysia Book of Records recognition awards.

#### Discussion.

*Diplodiscus latifii* is an endemic, rare tree. It is known only from one collection from Silam, Lahad Datu, Sabah. The newly-described species is morphologically most similar to *D. longifolius* in having an entire leaf margin, rounded midrib, terminal and axillary inflorescences with 3–4(–5)-flowered cyme-like units, bell-shaped calyx, short cylindrical androgynophore and densely stellate-lepidote ribbed fruits. However, *D. latifii* can be easily distinguished from *D. longifolius* by the morphological characters in Table 1.

**Table 1.** Comparison of diagnostic characters between *Diplodiscus latifii* and *D. longifolius*.

Character	*D. latifii*	D. longifolius*
Twig colour	Dark brown to black	Grey to light brown
Leaf		
Shape	Elliptic to broadly elliptic	Narrowly elliptic or obovate
Texture	Coriaceous	Chartaceous
Indumentum below	densely covered in brown dentate-lepidote scales with scattered dark brown subentire-lepidote scales (Fig. 2A, B)	densely covered in light brown stellate-lepidote scales (Fig. 2D, E)
Base	Subcordate	Cordate
Apex	Acute	Acuminate
Lateral veins	Prominent on both surfaces	Prominulous on both surfaces
Tertiary veins	Prominent on both surfaces	Faint on both surfaces
Petiole indumentum	densely brown dentate-lepidote scales (Fig. 2C)	densely light brown stellate-lepidote to glabrescent (Fig. 2F)
Flower bud		
Shape	Ellipsoid	Spheroid
Size (mm)	4–5 × 3.5–4	2–3(–5) × 2–3
Petal width (mm)	ca. 2.5	ca. 4.5
Ovary shape	Ellipsoid	Oblate
Fruit		
Shape	Ellipsoid	Broadly ellipsoid
Size (mm)	6–10 × 4–7	12–20 × 10–15

*Specimens examined: BORNEO. Sabah: Tawau – *A.D.E. Elmer 21312* (isotypes: BISH!, BO!, BM!, CAS!, CM!, HBG!, K!, L!, M!, MO!, SING!, UC!), Tanjung FR – *W.J. Pereira SAN 43717* (SAN!); Lahad Datu: *J. Bousi SAN 119318* (SAN!), Silam – *A. Agam SAN 37171* (K!, SAN!), *H.T. Sinanggul SAN 57445* (A!, SAN!), *R.P. Binideh SAN 55173* (K!, SAN!), Bakapit – *Heya & U. Juali SAN 61675* (K!, SAN!, L!), Pulau Sakar – *M. Chai SAN 26663* (K!, SAN!, L!), *G. Aban SAN 54818* (SAN!). Sarawak: Kuching – *J.D. Mamit S 32622* (L!, SAR!).

Morphologically, *Diplodiscus latifii* is also closely allied to the other four species found in Malaysia (i.e. *D. hookerianus*, *D. microlepis*, *D. parviflorus* and *D. scortechinii*) in having asymmetrical leaf bases, entire leaf margin, short petioles, lax panicles and united carpels. Nevertheless, this new species can be distinguished from these four species by its dark brown to black twigs, densely covered in brown dentate-lepidote scales with scattered dark brown subentire-lepidote scales below its elliptic to broadly elliptic leaves, prominent lateral and tertiary veins on both surfaces, basal pair of lateral veins ascending to ¼ of the blade length, larger flower buds greater than 2 mm in diameter, ellipsoid ovary and ribbed ellipsoid fruits. With the addition of this new species, there are now six species of *Diplodiscus* in Malaysia and a key to these species is provided below:

### Key to *Diplodiscus* species in Malaysia

**Table:** 

1	Leaves sparsely covered by stellate-lepidote scales below; petioles densely covered by subentire-lepidote scales.	***D. microlepis***
–	Leaves densely covered by stellate-, dentate- or subentire-lepidote scales below; petioles densely covered by stellate- or dentate-lepidote scales, stellate hairs or glabrescent.	**2**
2	Lateral veins 2–3 pairs with the basal pair ascending to ¾ of the blade length.	***D. scortechinii***
–	Lateral veins more than 5 pairs with the basal pair ascending between ¼ and ½ of the blade length.	**3**
3	Flower buds small, less than 2 mm in diameter; basal pair of lateral veins ascending to ½ of the blade length.	**4**
–	Flower buds large, more than 2 mm in diameter; basal pair of lateral veins ascending to ¼ of the blade length.	**5**
4	Leaf base rounded; lateral veins 7 pairs; panicles terminal.	***D. hookerianus***
–	Leaf base cordate; lateral veins 5–6 pairs; panicles terminal and axillary.	***D. parviflorus***
5	Leaves narrowly elliptic or obovate; densely covered in light brown stellate-lepidote scales below; acuminate leaf apex; flower buds spheroid, 2–3 mm in diameter; petals ca. 4.5 mm in diameter; ovary oblate; fruits broadly ellipsoid, 10–15 mm in diameter.	***D. longifolius***
–	Leaves elliptic to broadly elliptic; densely covered in brown dentate-lepidote scales with scattered dark brown subentire-lepidote scales below; acute leaf apex; flower buds ellipsoid, 3.5–4 mm in diameter; petals ca. 2.5 mm in diameter; ovary ellipsoid; fruits ellipsoid, 4–7 mm in diameter.	***D. latifii***

## Supplementary Material

XML Treatment for Diplodiscus latifii
